# Effective lipid extraction from undewatered microalgae liquid using subcritical dimethyl ether

**DOI:** 10.1186/s13068-020-01871-0

**Published:** 2021-01-09

**Authors:** Quan Wang, Kazuyuki Oshita, Masaki Takaoka

**Affiliations:** Department of Environmental Engineering, Graduate School of Engineering, Kyoto University, Cluster C, Kyoto Daigaku-Katsura, Nishikyo-ku, Kyoto, 615-8540 Japan

**Keywords:** Lipids, DME, Liquid–liquid subcritical extraction, Fames profile, Trace metals

## Abstract

**Background:**

Recent studies of lipid extraction from microalgae have focused primarily on dewatered or dried samples, and the processes are simple with high lipid yield. Yet, the dewatering with drying step is energy intensive, which makes the energy input during the lipid production more than energy output from obtained lipid. Thus, exploring an extraction technique for just a thickened sample without the dewatering, drying and auxiliary operation (such as cell disruption) is very significant. Whereas lipid extraction from the thickened microalgae is complicated by the high water content involved, and traditional solvent, hence, cannot work well. Dimethyl ether (DME), a green solvent, featuring a high affinity for both water and organic compounds with an ability to penetrate the cell walls has the potential to achieve this goal.

**Results:**

This study investigated an energy-saving method for lipid extraction using DME as the solvent with an entrainer solution (ethanol and acetone) for flocculation-thickened microalgae. Extraction efficiency was evaluated in terms of extraction time, DME dosage, entrainer dosage, and ethanol:acetone ratio. Optimal extraction occurred after 30 min using 4.2 mL DME per 1 mL microalgae, with an entrainer dosage of 8% at 1:2 ethanol:acetone. Raw lipid yields and its lipid component (represented by fatty acid methyl ester) contents were compared against those of common extraction methods (Bligh and Dryer, and Soxhlet). Thermal gravimetry/differential thermal analysis, Fourier-transform infrared spectroscopy, and C/H/N elemental analyses were used to examine differences in lipids extracted using each of the evaluated methods. Considering influence of trace metals on biodiesel utilization, inductively coupled plasma mass spectrometry and inductively coupled plasma atomic emission spectroscopy analyses were used to quantify trace metals in the extracted raw lipids, which revealed relatively high concentrations of Mg, Na, K, and Fe.

**Conclusions:**

Our DME-based method recovered 26.4% of total raw lipids and 54.4% of total fatty acid methyl esters at first extraction with remnants being recovered by a 2nd extraction. In additional, the DME-based approach was more economical than other methods, because it enabled simultaneous dewatering with lipid extraction and no cell disruption was required. The trace metals of raw lipids indicated a purification demand in subsequent refining process.
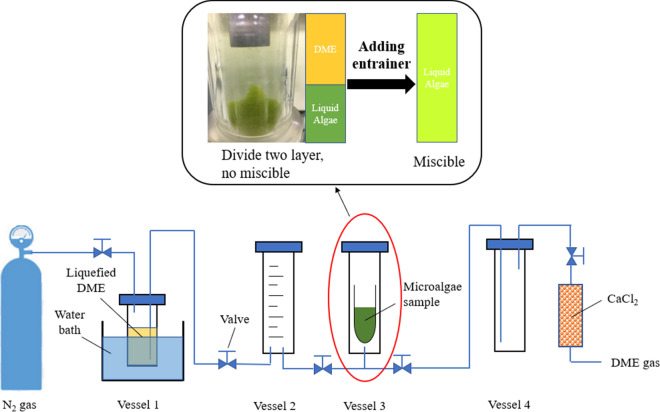

## Background

The development of renewable energy technologies has advanced in response to an ever-increasing demand for fossil fuels and global warming caused by CO_2_ emissions from their combustion [1–3]. Biodiesel produced from microalgae is considered a promising substitute for fossil fuels [4, 5] because microalgae boast a high growth rate and high lipid content. These features enable a high rate of carbon fixation with the potential for highly efficient biodiesel production. Furthermore, microalgae can be easily grown on non-arable land, which avoids competition with food production [6–8].

Biodiesel production from microalgae consists primarily of microalgae cultivation, harvesting, lipid extraction, and transesterification [9]. However, harvesting and lipid extraction are substantial bottlenecks in the development of an energy-efficient and cost-effective process for conversion of microalgae to biodiesel [10, 11]. Microalgae typically exhibit small cell size (5–50 μm) and low density (0.5–5 g/L) in growth media. These factors make it difficult to directly extract lipids without some form of harvesting pretreatment [12, 13]. In a typical process, microalgae suspensions are first thickened via gravity sedimentation, flocculation, or flotation to obtain slurry with a biomass content of 3–7%. This concentrated slurry is then mechanically dewatered by filtration or centrifugation to obtain cake with a biomass content of 10–25%. In a final step, the cake is thermally dried to a biomass content of > 90% [5, 14, 15].

Extraction of lipids is simpler from a completely dry microalgae sample than from a sample that has just been dewatered. A Soxhlet extraction with n-hexane obtained a 45% (dry base) yield of lipid from dried *Schizochytrium limacinum* [16]. Jie et al. [17] used ethanol to extract 48% (dry base) lipid from dried *Synechocystis PCC 6803*. Supercritical extraction using CO_2_ and ethanol has been used to obtain a 34% (dry base) yield of lipid from dried *S. limacinum* powder [16]; an 18.1% (dry base) lipid yield from dried *Chlorella spp.* powder was obtained using a mixed extraction solvent of methanol:ethyl acetate at a volume ratio of 2:1 [18].

However, drying is an energy-intensive process. Removal of 1 kg of water from mechanically dewatered microalgae (~ 20% biomass) by thermal drying requires 3560 kJ of energy input, rendering the net energy balance negative [5]; thus, the energy output from the extracted biodiesel is less than the energy needed to produce the biodiesel [3].

Energy consumption during biodiesel production from thermally dried microalgae is nearly 4000-fold greater than energy consumption during biodiesel production from merely mechanically dewatered microalgae (~ 20% biomass; wet microalgae) [19]. Hence, a positive net energy balance can be achieved without thermal drying [3, 13]. Therefore, several investigations have focused on lipid extraction from wet microalgae. For example, Lakshmikandan [20] used a mixed solvent of hexane and isopropanol (3:2 v/v) to extract lipids from centrifugation-harvested *Chlorella vulgaris* (biomass content ~ 8.9%) and obtained a maximum lipid yield of 22.5% (dry base). Ethyl acetate has been used to extract lipids from wet *Isochrysis galbana* (5% biomass) at a yield of 17.6% [21]. Among six evaluated solvent systems, isopropanol:hexane (2:1 v/v) was the most effective in extraction of lipids from wet *Scenedesmus obliquus* (20% biomass), affording a 7.8% lipid yield (dry base) [3]. A solvent system of chloroform:methanol:sulfuric acid (1:1:0.05 v/v), combined with microwave irradiation, was used to obtain a 19.0% (dry base) yield of lipids from centrifuged *Chlorella pyrenoidosa* (water content, 80 wt%) [22].

Because dewatering via centrifugation or filtration requires considerable energy compared to the thickening process [23], direct extraction of lipids from thickened microalgae would significantly improve the net energy gain. Yet with the water content of microalgae increasing, more solvent may be needed to match the phase mass transfer equilibrium, and the cost for the increased solvent could be considerable; thus, it is necessary to achieve the above objectives without increasing solvent consumption. Liquid dimethyl ether (DME) is a promising solvent for extraction of lipids from thickened microalgae. Liquid DME is partially miscible with water (7–8 wt% DME; room temperature) and features a high affinity for organic compounds. Thus, DME is suitable for extraction of lipids from wet biomass samples with simultaneous dewatering. This combined process represents considerable energy savings [24, 25]. In addition, DME is a gas under ambient conditions; thus, it can be easily liquefied at 0.51–0.59 MPa and room temperature (20–25 °C). The low boiling point of DME (i.e., − 25 °C) allows it to be easily removed via evaporation for recycling/reuse [25, 26].

DME has been successfully applied for extraction of lipids and removal of moisture from dewatered biomass, including sludge [25, 27], cattle manure [28], microalgae [29] and vegetables [30]; however, its use with merely thickened samples has not been studied. This is largely because its low polarity results in immiscibility with microalgae suspended in water. Although DME can absorb a small fraction of water, as mentioned above, greater proportions of water result in discrete aqueous and organic layers, thereby preventing DME from coming into close contact with microalgae cells. This effect is more noticeable with marine algae, because the polarity of the algal slurry is enhanced by dissolved salts. Although not the most economical approach, one solution involves the use of large volumes of DME [31]; specifically, a 167:1 weight ratio of DME to microalgae (dry base) was necessary for extraction of lipids from a sample containing 9% solids. Another potential solution involves the use of a solvent with infinite miscibility in water for adjustment of DME polarity. Adjustment of the cosolvent ratio allows DME to be completely mixed with water. This type of additive is regarded as an entrainer, and has been shown to improve the efficiency of supercritical fluid extraction [32, 33].

The liquified DME also has been reported to be a kind of green solvent [34]. Very often, challenges with conventional solvents arise due to their toxicity and impact on the environment. And the DME received significant attention as an alternative solvent due to its nontoxic, environmentally friendly, easier to transport, potentially renewable and relatively cheap [35]. Even DME is safety for food industrials, since the residual DME present in food products is expected to be significantly below the lowest no-effect limit and in some cases below the detection limit which makes the extracted material potentially safe for consumption [36].

The novelty of this study was to develop a microalgal lipid extraction technology based on DME subcritical extraction, which was suited for undewatered samples. Since the subcritical extraction was realized, the operation condition (0.51–0.59 MPa, room temperature) was easier to be achieved with less operating costs and security risks than the supercritical fluid extraction (SCF) [37], while it retained SCF’s advantages (higher selectivity, lower extraction time, and non-requirement of a follow-up separation) [37]. And the DME method in this study also eliminated energy or chemicals consumption of additional cell disintegration usually used in other methods [38]. In addition, compared with the formerly reported DME method [29], this study modified it to be applied to undewatered microalgae (~ 95% of water content) and further reduced the consumption in the sample pretreatment, while the DME dosage was not increased. Furthermore, this technology made microalgae residues after lipid extraction the low moisture content by simultaneously dewatering/drying, and hence had a potential to be used as solid biofuel. This combined process represented considerable energy savings.

Here, liquefied DME was used to extract lipids from AlCl_3_-flocculated *Nannochloropsis oculata* (solid content 18.3 g/L). A suitable entrainer for DME was identified among ethanol, dimethyl sulfoxide (DMSO), acetone, and tetrahydrofuran (THF). Extraction performance was evaluated with respect to changes in extraction time, DME dosage, entrainer composition, and entrainer proportion in DME. The performance of our modified DME-based method was compared to the performances of Bligh and Dryer, and Soxhlet methods in terms of raw lipid yield, fatty acid yield, and C/H/N composition. For each method, extracted lipids were characterized by thermal gravimetry (TG)/differential thermal analysis (DTA), Fourier-transform infrared spectroscopy (FTIR), and trace elemental analyses.

## Results and discussion

### Entrainer screening for improved lipid extraction using DME

Figure 2a shows the yields of extracted raw lipids and FAMEs using our DME-based method (25 mL DME for 8 mL of microalgae) with each of the four entrainers. The blank system yielded 14.4 mg/g (dry base) raw lipid. In contrast, 57.3, 16.4, 91.8 and 16.3 mg/g raw lipid were obtained with 2.5 mL of ethanol, DMSO, acetone, and THF, respectively. These results showed that both ethanol and acetone were effective entrainers, enhancing raw lipid yields by factors of 4.0 and 6.4 relative to the blank, respectively. DMSO and THF were less effective, producing only ~ 1.1-fold improvements in raw lipid yields. The proportions of FAMEs obtained also varied among entrainers. For example, the highest proportion of FAMEs (62.0%) in the extracted raw lipid was obtained with the blank. Experiments incorporating THF yielded a similar value of 60.5%. The FAME proportions obtained using ethanol, DMSO, and acetone were 48.0%, 49.6%, and 41.4%, respectively. Notably, the yields of FAMEs (absolute value, Fig. 2a) obtained with ethanol (27.4 mg/g DB) and acetone (37.9 mg/g DB) were much higher than those obtained with the blank (8.9 mg/g DB), despite the smaller proportions. The solids contents (mainly containing microalgae cell residues and a small part of remaining salt) of microalgae samples after DME treatments using the four entrainers are shown in Additional file 1: Figure S1. The solids content of the blank increased to 19.4% after DME treatment of flocculation-thickened microalgae containing ~ 95% water. Thus, water and lipids were extracted simultaneously from microalgae samples using liquefied DME and an entrainer. Accordingly, this process can be regarded as an effective dewatering method. However, the addition of an entrainer did not enhance dewatering. The solids contents of the acetone and THF groups were 20.4% and 20.7%, which represented no significant improvements over the blank group. A slight reduction in solids content (17.2%) was observed when using ethanol as the entrainer. The solids contents of samples treated with DMSO were only 12.5%, demonstrating that DMSO was not an effective dewatering agent.

The FAME compositions of the various samples are shown in Additional file 1: Figure S2. No obvious differences were observed. For all five groups (blank and four entrainers), FAMEs in the extracted raw lipids were composed primarily of C16:0, C16:1, and C18:1n9c, representing 60–70% of the total FAMEs. The remaining FAMEs were distributed between C4:0 and C24:1n9. These findings indicated that the entrainer did not affect the FAME composition of the extracted raw lipids.

Given the above results, ethanol and acetone, each of which significantly improved lipids yields with minimal effects on dewatering, were selected for further investigation as entrainers. DME has a low polarity (dielectric constant of 5.02) and is generally immiscible with water (dielectric constant of 78.54). However, hydrogen bonds formed between DME and water can result in partial miscibility [39], enabling DME to efficiently absorb water from microalgae [29]. In the present study, the amount of water in microalgae samples exceeded the miscibility limit of DME, which can only absorb 7–8 wt% water. Therefore, our mixtures of DME and microalgae separated into two liquid layers, preventing the close contact between DME and algal cells that is required for lipid extraction. The addition of ethanol (dielectric constant of 24.60) or acetone (dielectric constant of 21.01) changes the polarity of the organic phase [40], increasing its miscibility with water. Conversely, the addition of THF (dielectric constant of 7.52) did not improve lipid extraction because it precipitated out of the microalgae slurry (sea water) [41]. Among the four entrainers, DMSO had the highest dielectric constant of 47.00, implying good solubility in water and poor compatibility with DME. This latter feature explains the failure of DMSO to improve lipid extraction. Thus, the dielectric constant of an ideal entrainer should be neither excessively high nor excessively low.

### DME-based extraction with an ethanol–acetone entrainer

**Fig. 1:**
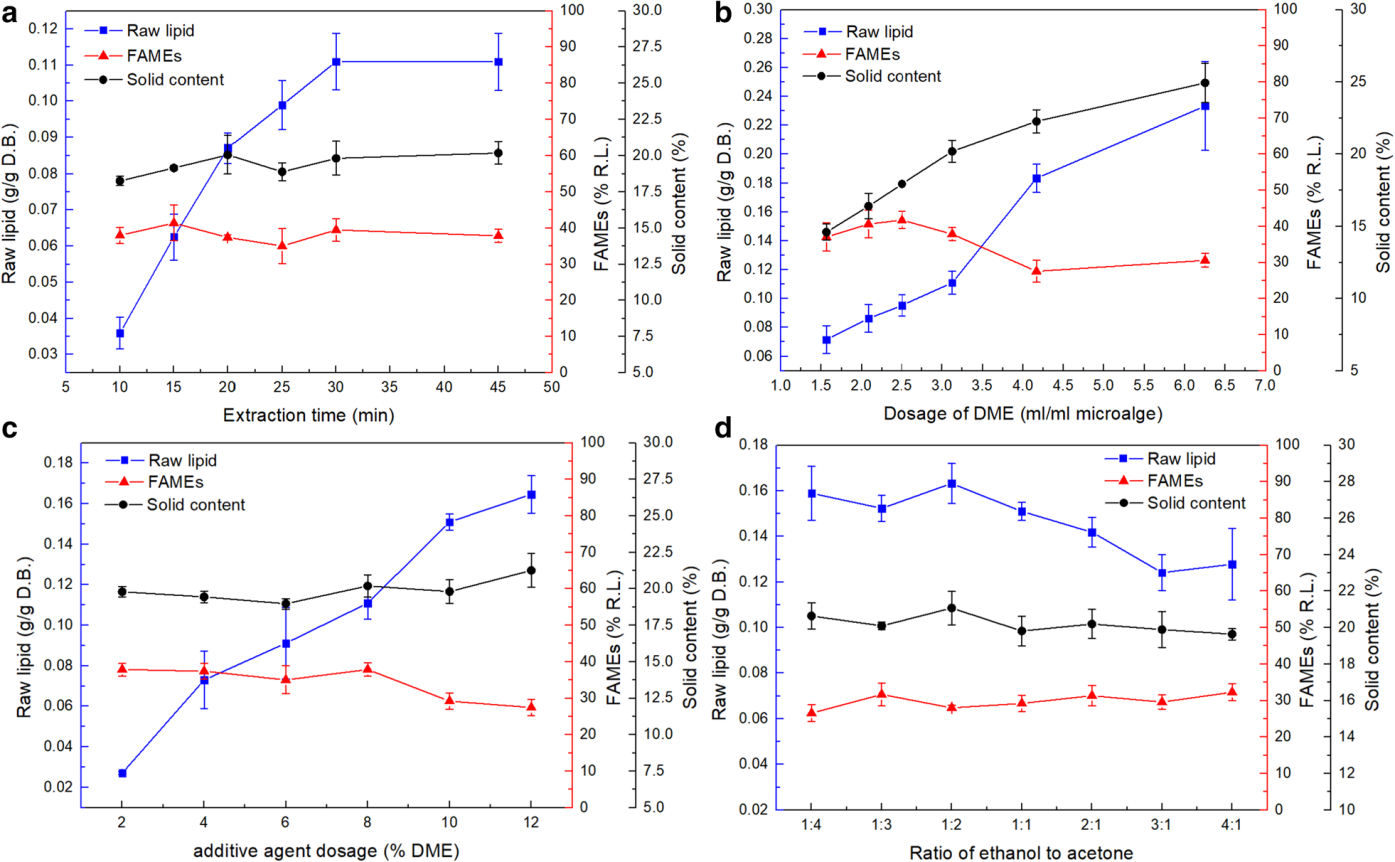
Lipid extraction and dewatering using our DME-based method with an entrainer of ethanol-acetone. Influences of **a** extraction time, **b** DME dosage, **c** entrainer dosage, and **d** ethanol:acetone ratio on raw lipids yield are shown

The effects of extraction time on the lipid yields using our DME-based method are shown in Fig. 1a. Raw lipid yields increased with extraction time, reaching 0.0359 g/g dry biomass after 10 min, 0.0871 g/g dry biomass after 20 min, and 0.1110 g/g dry biomass after 30 min. No further increases in yield were observed beyond 30 min. The proportions of FAMEs in the extracted raw lipids ranged from 35.0 to 41.1%. Changes in FAME proportions as a function of extraction time were not obvious. Similarly, the solids content of microalgae after extraction remained consistent between 10 and 45 min. The lowest solids content of 18.3% was observed after 10 min of extraction; this increased to 20.2% after 45 min. Thus, only 30 min was required to fully extract the raw lipids. Furthermore, the rates of FAME and non-FAME transfer to DME were similar. Therefore, dewatering by DME-based extraction could be accomplished in a relatively short time (10 min).

Figure 1b shows the effects of DME dosage. Raw lipid yield increased dramatically with the dosage of DME. Using 1.6 mL DME/mL microalgae yielded 0.0716 g raw lipid/g dry biomass. A DME dosage of 6.3 mL/mL yielded 0.2333 g raw lipid/g dry biomass. At DME dosages as high as 6.3 mL/mL, the trend of increasing raw lipid yields with increasing DME dosage did not diminish. The proportions of FAMEs obtained with DME dosages of 1.6, 2.1, 2.5 and 3.1 mL/mL were 37.1%, 40.6%, 41.8%, and 37.9%, respectively. Thus, no significant differences in FAME proportions were observed as a function of DME dosage. However, when the DME dosages were increased to 4.2 and 6.3 mL/mL, the proportions of FAMEs decreased to 27.6% and 30.6%, respectively. This effect can be attributed to diminishing returns. FAMEs become more difficult to extract as their concentrations in microalgae decrease. In contrast, residual non-FAME components were present; thus, the diminishing effect was less obvious. This hypothesis will be discussed in Sect. "Raw lipid yields and FAME profiles". Analysis of the solids contents of various samples after extraction revealed that water removal increased with DME dosage. At a DME dosage of 1.6 mL/mL, the solids content of the microalgae was 14.6%; it increased to 24.9% with 6.3 mL/mL DME. Increasing the DME dosage significantly improved raw lipid extraction with simultaneous dewatering. However, the reduced proportions of FAMEs at DME dosages exceeding 4.2 mL/mL indicated a weakening effect at higher dosages.

Extraction performance as a function of entrainer dosage is illustrated in Fig. 1c. Raw lipid yield grew nearly linearly with increasing entrainer dosage (ethanol + acetone). An entrainer dosage of 2% (in DME) slightly enhanced raw lipid yield to 0.0271 g/g dry biomass. At a dosage of 6%, the yield was 0.0912 g/g; a dosage of 12% entrainer yielded 0.1646 g/g raw lipids. Within an entrainer range of 2%–8%, the amounts of extracted FAMEs (at a constant proportion of ~ 37.1%) increased with increasing entrainer dosage. However, FAME proportions decreased to 27.4% at an entrainer dosage of 12%. The effects on dewatering were minimal, and the observed solids content remained stable at 19.9%. Thus, FAME proportions decreased at entrainer dosages exceeding 10%, while raw lipid yields continued increase. As a result, the benefit to FAME extraction by increasing the entrainer dosage from 8 to 12% was limited; 8% or 10% entrainer was suitable for the DME-based extraction of FAMEs.

The influence of ethanol:acetone ratio on lipid yield is shown in Fig. 1d. Higher raw lipid yields were obtained at lower ratios of ethanol to acetone. Differences between lipid yields obtained at ratios of 1:4 to 1:2 were minimal, with yields of approximately 0.1581 g/g. Raw lipid yield began to decrease at a ratio of 1:1; yields of 0.1241 and 0.1278 g/g were obtained for ratios of 3:1 and 4:1, respectively. The effects of this ratio on FAME proportions and solids contents were not obvious; they ranged from 26.5 to 32.3% and from 19.6 to 21.1%, respectively. The greatest yields of both raw lipids and FAMEs were obtained at relatively low ratios (1:3 or 1:2) of ethanol to acetone.

Generally, raw lipids extracted from microalgae contain non-polar lipids (e.g., triglyceride, wax esters, and steryl esters), polar lipids (e.g., phospholipid and glycolipid), free fatty acids, sterols, proteins, hydrocarbons, pigments, and other algal products [1, 42, 43]. Because these components differ in polarity, the extraction process of each is influenced by solvent polarity. Shin et al. [44] used various types of solvents to extract lipids from microalgae; they observed the lowest lipid yield with hexane, a non-polar solvent. In contrast, more lipids were extracted by a medium polarity solvent, such as isopropanol. The greatest number of lipids was extracted using a mixture of hexane and isopropanol. The advantages of combining non-polar and polar solvents in the extraction of lipids have been demonstrated in previous reports [3, 45]. In this study, the low polarity of DME allowed strong interactions with neutral lipids. The addition of ethanol and acetone enhanced interactions with polar lipids. This effect is ultimately responsible for the enhanced raw lipid yields and FAME proportions described above. Water in flocculated microalgae acts as a barrier between intracellular lipids in cells and DME [6, 40]. The miscibility of entrainers with water was crucial to achievement of efficient lipid extraction with minimal DME consumption. In addition, the solids contents of samples increased to ~ 20% after DME treatment, indicating a simultaneous dewatering effect.

### Comparison of DME-based extraction using an entrainer with Bligh and Dryer, and Soxhlet  extraction methods

#### Raw lipid yields and FAME profiles

**Fig. 2:**
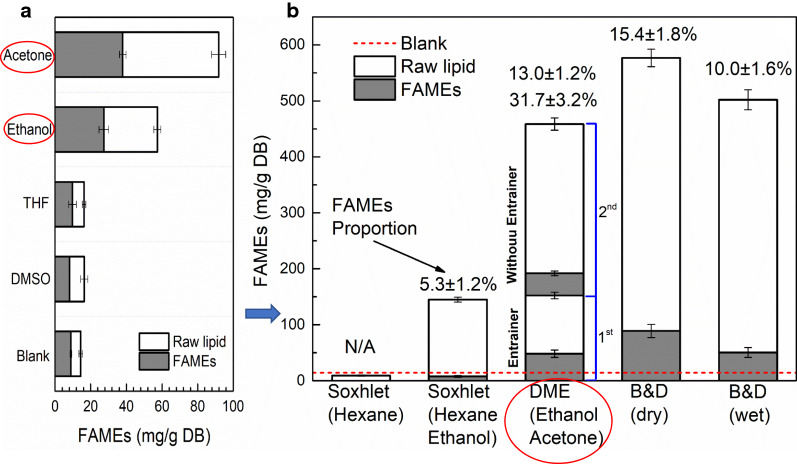
Raw lipid yields with FAMEs **a** entrainer screening, **b** comparison with conventional methods

Lipid extraction using our DME-based method was compared with Bligh and Dryer, and Soxhlet extractions, as shown in Fig. 2b. The B&D method resulted in the highest raw lipid yields; extraction from dry and wet samples achieved 576.6 and 502.2 mg/g dry biomass, respectively. Our DME method extracted 152.2 mg raw lipid per 1 g microalgae (dry base) during the first extraction process, which was similar to the yield obtained with a Soxhlet extraction using HE (144.9 mg/g), while the hexane-based Soxhlet extraction hardly extracted lipids. It should be noticed that, due to the simultaneous dewatering effect by DME, water content of microalgae reduced, and lipid became easier to extract after the first DME extraction process. Thus, 306.5 mg/g raw lipid was extracted by 2nd extraction process of DME even without any entrainer addition.

**Fig. 3:**
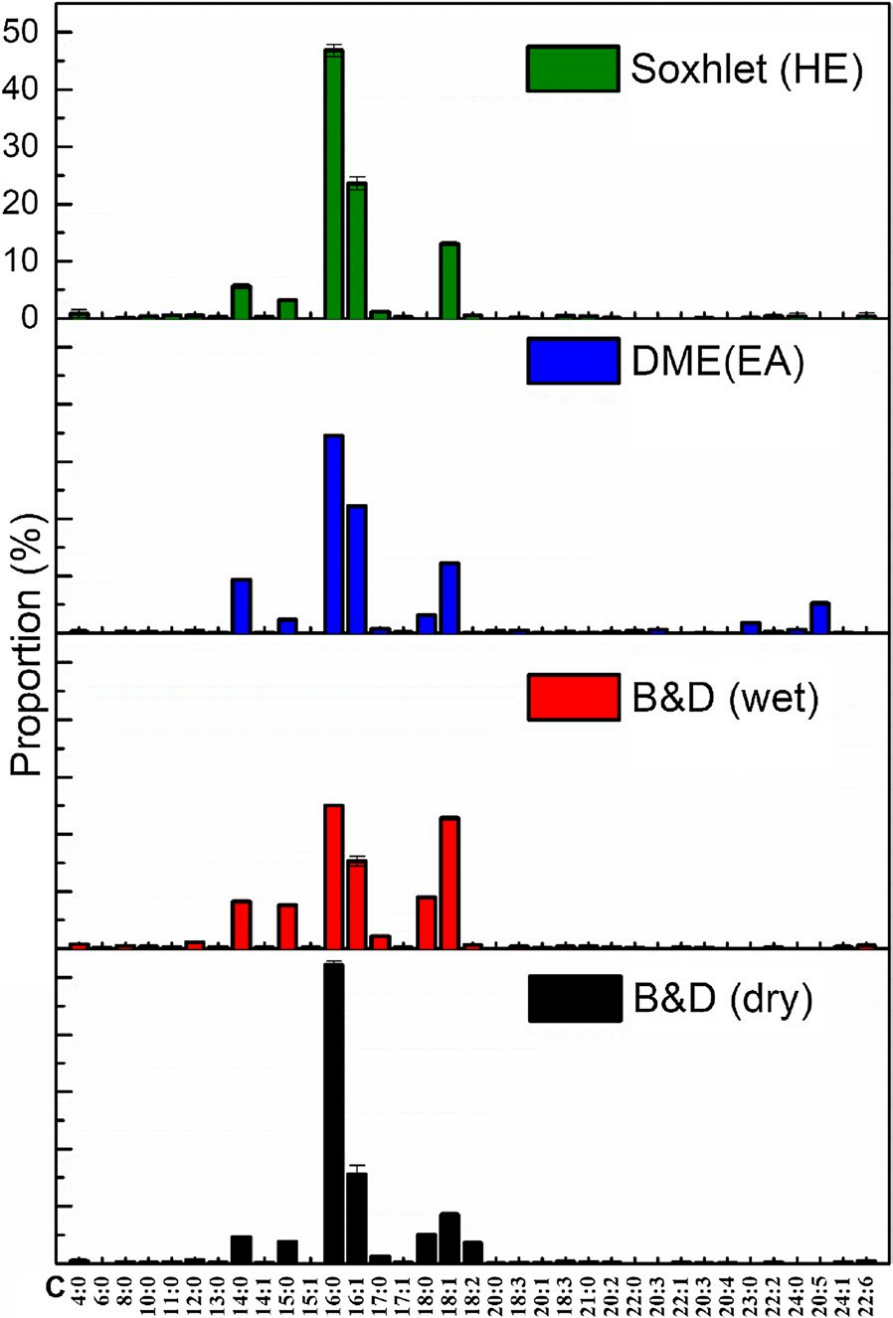
FAME profiles obtained by four extraction methods

The relatively highest proportion of FAMEs was obtained using the DME method (31.7% of 1st process, 13.0% of 2nd process), followed by 15.4% with B&D (dry), 10.0% with B&D (wet), and 5.3% with Soxhlet extraction (HE). These values are consistent with the findings in previous studies [25]. In addition, significant differences were observed in FAME profiles among extraction methods, as shown in Fig. 3. All evaluated methods yielded FAME profiles containing primarily C14:0, C16:0, C16:1, C18:0, C18:1, and C18:2, which constituted 81.1–89.8% of the total FAMEs. However, the relative amount of each lipid varied among methods. For example, C16:0 constituted 52.2% of the FAMEs obtained via B&D (dry), while the fraction of C16:0 obtained by extraction via Benchmark B&D (wet) was 25.0%. Relatively higher C16:1 proportions were obtained by DME and Soxhlet (HE) extraction (22.2% and 23.6%, respectively), compared to B&D (dry) with B&D (wet) extractions (~ 15%). The proportion of C18:1 by B&D (wet) was 22.87%, much higher than the proportion achieved with other methods. Thus, compared to Soxhlet (HE) extraction, DME method shows clear advantages for the extraction of FAMEs. The addition of ethanol into hexane did not make the solvent mixture miscible with water; thus, contact between hexane and lipids in the microalgae could not be achieved. However, certain non-lipids (e.g., sugars and pigments) were extracted into the ethanol–water phase and transferred to the HE phase, comprising the extracted raw lipid.

When B&D (dry) extraction is regarded as the benchmark for total lipid recovery [29, 46], DME method of 1st extraction process extracted 26.4% of the total raw lipids and 54.4% of the total FAMEs in the microalgae. Therefore, the DME preferred to extract lipid component (represented by FAME) rather than non-lipid part; otherwise, the ratio of total raw lipids and FAMEs being extracted should be the same. Although the process did not achieve complete lipid extraction, simultaneous dewatering would allow the remaining lipids to be easily recovered in the second DME extraction process, by which 53.2% of total raw lipids with 44.7% of the total FAMEs was further extracted by the 2nd process. Here, this second extraction demonstrated the diminishing returns mentioned in 2.2. Since the lipid component was preferentially extracted, FAME yields easily reached its limit with the increasing of DME dosage (1st extraction process reached to 54.4% and 2nd extraction process accumulated to 99.1%) and that increase rate of FAME yields slowed down with the further increasing of DME dosage was observed. Unlike it, after the 2nd extraction, there was still 20.4% of the total raw lipid remaining (it was almost all non-lipid component) and this diminishing returns on the total raw lipid was hence not obvious. Moreover, the 2nd extraction process was not indispensable, since more than half of FAMEs has been extracted in the 1st process and it is acceptable to sacrifice some extraction efficiency in exchange for low cost. The DME method achieved lipid extraction from liquid-like microalgae sample; the energy and materials cost for dewatering samples in conventional methods can be saved. Because DME is easily recycled and reused [25], DME extraction can be considered highly economical. In addition, the DME method in this study did not require cell disruption [1] via ultrasound or microwave irradiation, high-pressure homogenization, enzymatic hydrolysis, or chemical hydrolysis, while cell disruption by one or more of these processes is considered an indispensable pretreatment in other lipid extraction methods.

#### FTIR, TG/DTA, and elemental composition

**Fig. 4:**
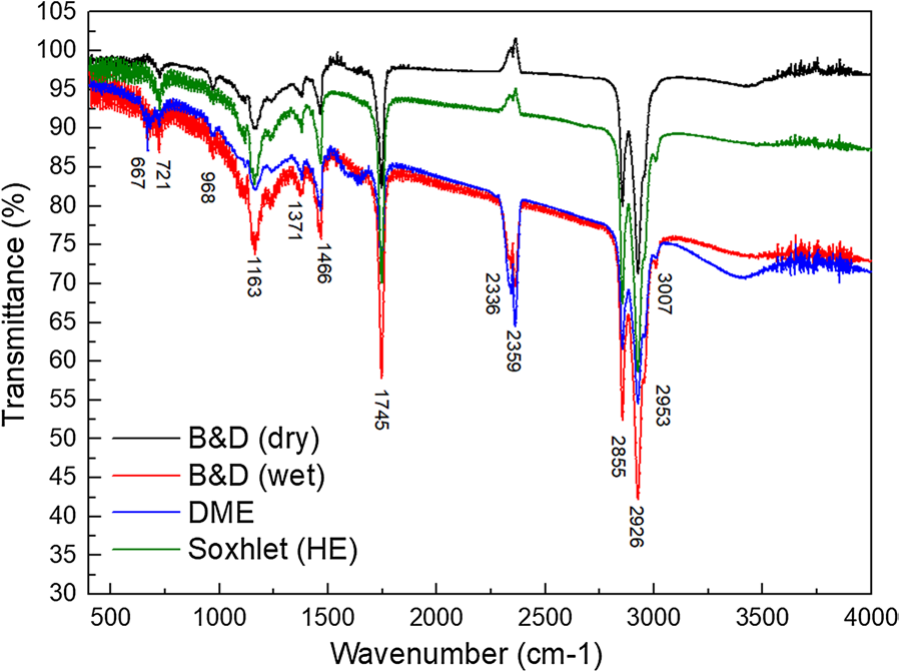
FTIR spectra of lipids obtained using the four extraction methods

FTIR spectra of extracted raw lipids are shown in Fig. 4. The peaks at 3007 cm^−1^ were consistent with olefinic C–H stretching in unsaturated fats [47]. The peaks at 2953 cm^−1^ were assigned to C–H stretches in –CH_3_. The strong absorption peaks at 2926 and 2855 cm^−1^ corresponded to C–H stretches in –CH_2_. The double peaks at 2359 and 2336 cm^−1^ corresponded to N–H stretching vibrations of amino groups [48, 49]. The C=O stretch of an ester could be observed at 1745 cm^−1^. Symmetric and asymmetric bending of methyl groups resulted in peaks at 1371 and 1466 cm^−1^, respectively; the C–O ester stretch was evidenced by a peak at 1163 cm^−1^. Peaks at 968 cm^−1^ corresponded to stretching vibrations of the C–C backbone. The lipid spectra were similar, regardless of the extraction method. The only exception was that N–H peaks (2359, 2336 cm^−1^) from samples obtained by B&D (wet) and DME methods were much stronger than those obtained by B&D (dry) and Soxhlet (HE) methods. These findings indicated that raw lipids extracted by B&D (wet) and DME methods contained greater levels of nitrogen.

**Fig. 5:**
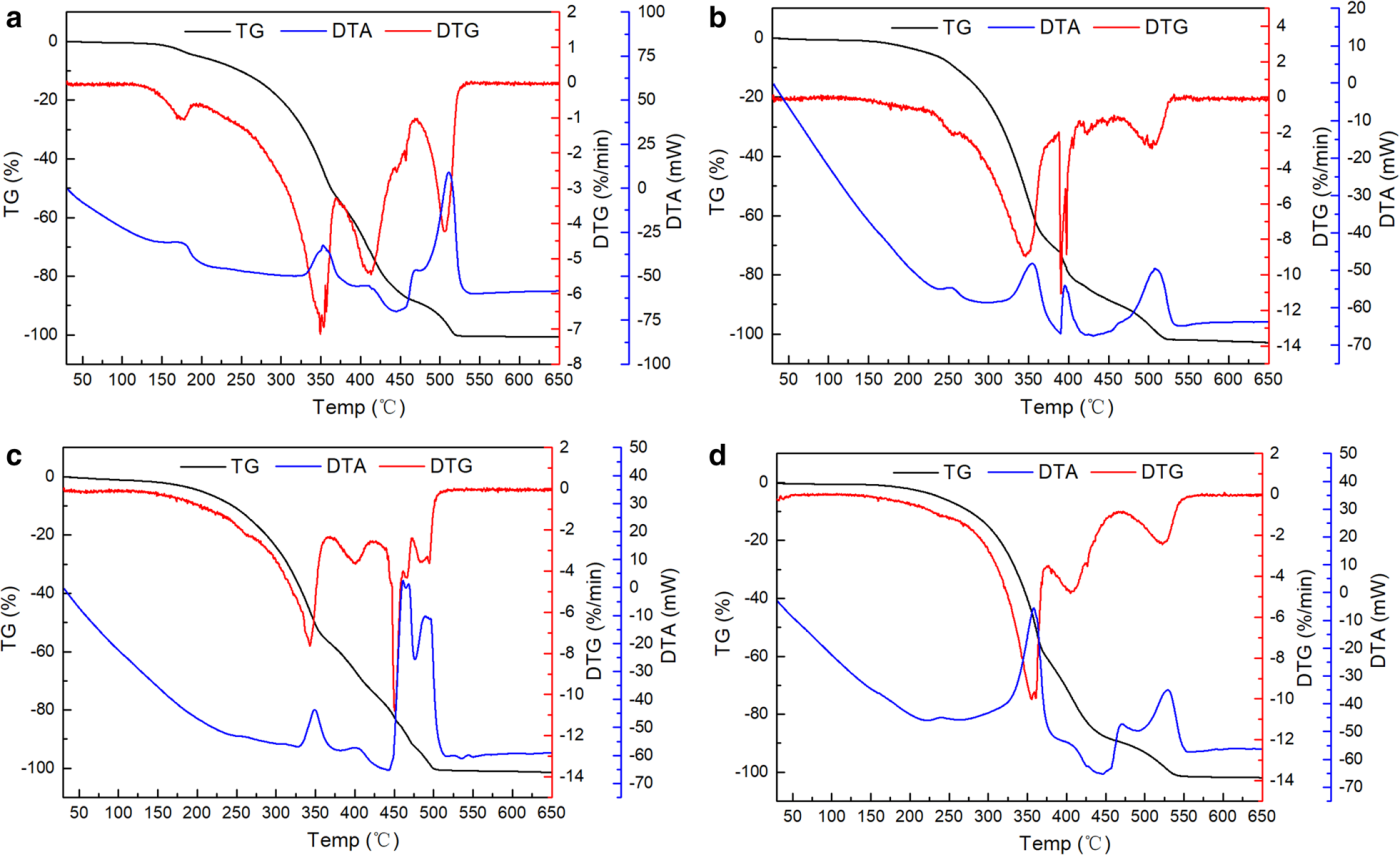
TG/DTA thermograms of raw lipids obtained via **a** B&D (dry), **b** B&D (wet), **c** DME, and **d** Soxhlet (HE) extraction processes

The oxidative and thermal stabilities of extracted raw lipids are shown in Fig. 5. For each of the four raw lipid samples, mass loss could be roughly divided into three stages [50, 51]: ~ 50% of the mass loss occurred during the first stage with a derivative thermogravimetry peak and corresponding DTA exothermic peak at 350 °C. This transition is due to the formation of alkyl radicals and their reaction with oxygen. Unsaturated lipids decomposed in this stage. In the second stage (exothermic DTA peak), a ~ 20% loss of mass occurred with a derivative thermogravimetry peak at 400–415 °C. This can be attributed to the degradation of carbon chains and decomposition of any remaining unsaturated lipids and saturated lipids. The last exothermic stage, from 450 to 550 °C, corresponded to the complete oxidation of carbonaceous residues from the first two stages. Slight differences were observed in the thermal data of the four lipid samples. A small derivative thermogravimetry peak (~ 3% mass loss) at 175 °C was present in the thermogram of raw lipids obtained from B&D (dry). This exothermic peak can be attributed to the initial degradation of esters by the action of oxygen, rather than the evaporation of lipids [52].

Additional file 1: Table S2 shows the C/H/N compositions of the extracted raw lipids. Nitrogen content is an important metric when using extracted lipids as biofuel; low N content is preferable for combustion [53]. In this study, the N contents in extracted lipids ranged from 0.09 to 0.23%, among the various methods. The highest N content of 0.23% was obtained by DME, which was consistent with the FTIR data. The B&D (wet) extraction yielded lipids relatively high in C (76.21%) and low in O (11.26%). This can be attributed to the isolation of a wide range of components from microalgae [29].

#### Trace metal analyses

Trace metals in biodiesel can lead to metallic corrosion during transport and storage, enhanced pollutant emission, and engine deterioration [54, 55]. Relative to the blank (ultrapure water), trace inductively coupled plasma mass spectrometry analyses indicated the presence of Fe, Ni, Cu, Zn, Sr, Ba, Mg, K, and Na in the extracted raw lipids, as shown in Table 1. Ca and Si, which were reported by Kanda et al. [29], were not found in our microalgae samples. Trace metals in this study were derived primarily from the cultivation broth. The concentrations of Mg, Na, K, and Fe were particularly high. The Mg content in samples extracted using our DME-based method was 66‱. This indicates that further purification would be required to remove trace metals before using the extracted materials as vehicle fuel. Raw lipids extracted using the B&D (wet) method will likely require a similar treatment; the sum of Fe, Mg, and Na contents was 19‱.

**Table 1:** Trace metals in extracted raw lipids

		Fe	Ni	Cu	Zn	Sr	Ba	Mg	K	Na
		(‱)	(‱)	(‱)	(‱)	(‱)	(‱)	(‱)	(‱)	(‱)
B&D (dry)	Mean	0.416	0.029	0.049	0.041	0.326	0.200	2.067	–	4.500
	Variance	0.015	0.001	0.003	0.007	0.001	0.002	0.173	–	0.929
DME	Mean	0.824	0.075	0.746	0.353	0.005	0.013	66.633	1.867	1.833
	Variance	0.023	0.002	0.011	0.018	0.000	0.002	0.416	0.000	0.100
Soxhlet (HE)	Mean	0.380	0.044	0.030	0.056	0.015	0.067	3.067	–	–
	Variance	0.011	0.001	0.001	0.008	0.000	0.003	0.520	–	–
B&D (wet)	Mean	3.334	0.388	0.082	0.173	0.042	0.043	7.933	0.533	7.867
	Variance	0.056	0.006	0.010	0.015	0.001	0.001	0.058	0.115	0.115

## Conclusions

A mixture of ethanol and acetone was used as an entrainer to significantly improve the yield of lipids extracted from flocculation-harvested microalgae with DME. Addition of ethanol or acetone to DME enhanced its miscibility with water and improved lipid yields by factors of 4.0 and 6.4, respectively. Optimal conditions for lipid extraction were: 30 min, 4.2 mL DME per 1 mL microalgae, and an entrainer dosage of 8% at an ethanol:acetone ratio of 1:2. Under these optimal conditions, a comparison of our DME method (entrainer) against conventional B&D and Soxhlet extraction methods revealed that the DME method of 1st process extracted 26.4% of the total raw lipids with 54.4% of the total FAMEs in microalgae, and remnants could be easily recovered by a 2nd extraction process. Among all evaluated methods, the proportion of FAMEs was highest using our DME-based method. Finally, the extracted raw lipids obtained by each method were characterized by thermal analyses and FTIR spectrometry. Enhanced nitrogen levels in lipids extracted by B&D (wet) and our DME-based method, as indicated by strong N–H vibrations in FTIR spectra, were confirmed by C/H/N analyses. TG/DTA data indicated that extracted lipids began to decompose at 150 °C, with the final decomposition temperature varying from 500 to 535 °C. Inductively coupled plasma analyses showed traces of Fe, Ni, Cu, Zn, Sr, Ba, Mg, K, and Na in the extracted raw lipid samples. DME-based extraction resulted in particularly high levels of Mg in the produced lipids, indicating that further purification would be required prior to the use of these extracted lipids as biofuel.

## Materials and methods

### Microalgae cultivation and pretreatment

Marine microalgae strain *N. oculata* was obtained from the Microbial Culture Collection at the National Institute for Environmental Studies (Tsukuba, Japan). The strain is a genus of unicellular, oval-shaped, and non-mobile marine microalgae with a cell diameter of 3–8 μm. The algae were cultivated in ESM medium [56], as shown in Additional file 1: Table S1, in 20-L bucket photo-bioreactors at 20 ± 2 °C with light/dark cycles of 12 h/12 h. After 2 weeks of cultivation, the microalgae entered the stationary phase and were thickened by induced flocculation using 60 mg/L AlCl_3_.

The biomass concentrations of microalgae before and after flocculation were measured. Samples were filtered through a vacuum filter with pre-weighted glass filter paper (GF/C, 1.2 um, 4.7 cm; Whatman plc, MA, USA) and were washed several times with deionized water. The filtrate was dried in an oven at 105 °C for 24 h to achieve a constant weight and cooled to room temperature in a desiccator prior to weighing. This process was repeated in triplicate. The biomass concentration of the original microalgae was 0.64 ± 0.01 g/L; it increased to 18.3 ± 0.79 g/L following flocculation. Thus, the flocculated samples for the lipid extraction were composed of water (948.3 g/L), microalgae (18.3 g/L D.W.) and salts (33.4 g/L, from cultivation medium of sea water, Additional file 1: Table S1).

### DME extraction

#### Device description and experimental procedure

As shown in graphical abstract, the DME extraction system was composed of five parts: a liquefied DME storage vessel (vessel 1, TVS-1-100, 500 cm^3^, Taiatsu Techno Corp., Saitama, Japan), a vessel to measure DME (vessel 2, 100 cm^3^, HPG-96-3, 26.5 mmφ × 238 mm, Taiatsu Techno Corp.), a vessel for lipid extraction (vessel 3, 100 cm^3^, HPG-96-3, 26.5 mmφ × 238 mm, Taiatsu Techno Corp.), a vessel for separating liquid from solvents (vessel 4, 100 cm^3^, HPG-96-3, 26.5 mmφ × 238 mm, Taiatsu Techno Corp.), and a moisture trap column of CaCl_2_ (HPG-10–5, 11.6 mmφ × 190 mm, Taiatsu Techno Corp.).

Flocculation-thickened microalgae were loaded onto an extraction column containing a cellulose extraction thimble (glass fiber, φ25 × 100 mm, Whatman, Maidstone, United Kingdom). The entire assembly was placed in vessel 3 after the entrainer had been added. A specified amount of liquefied DME was pushed from vessel 1 to vessel 2 by N_2_ gas (ZERO-A, Sumitomo Seika Chemicals Company, Ltd., Japan). The measured volume of DME in vessel 2 was then allowed to flow into vessel 3. After several minutes of extraction, only the liquid phase in vessel 3 was transferred to vessel 4. DME in the liquid evaporated with pressure relief and the raw lipids were deposited on the upper surface of the residual liquid (entrainer, water, and salts). The separated raw lipids were dissolved in 5.0 mL of chloroform (Guaranteed Reagent, Wako Co., Ltd.) and filtered through a 0.45-μm membrane filter (DISMIC-13HP, Advantech Co., Ltd., Japan). The chloroform was then removed by a stream of nitrogen gas (Nitrogen Termovap Sample Concentrator, Tokyo Rikakikai Co., Ltd., Japan); the recovered raw lipid concentrate was weighed and stored below 0 °C.

#### Entrainer screening

Four potential entrainers (Table 2), miscible in water at any ratio, were evaluated for their efficiency in lipid extraction using liquid DME. In each experiment, 25 mL of DME was used to extract the lipids from 8 mL of flocculation-thickened microalgae (18.3 ± 0.79 g/L) over 30 min. As an entrainer, 2.5 mL of ethanol, DMSO, THF, or acetone (Guaranteed Reagent, Wako Co., Ltd.) was added. The results were compared with those obtained with a blank mixture that did not include an entrainer.

**Table 2:** Characteristics of solvents used in this study

	Water	DME	Entrainers			
			Ethanol	DMSO	THF	Acetone
Formula	H_2_O	C_2_H_6_O	C_2_H_6_O	C_2_H_6_OS	C_4_H_8_O	C_3_H_6_O
Molecular weight	18.02	46.07	46.07	78.13	72.11	58.08
Density (g/mL)	0.998	1.970	0.789	1.092	0.883	0.785
Melting point (°C)	0.0	− 138.0	− 114.1	18.4	− 108.4	− 94.7
Boiling point (°C)	100.0	− 25.0	78.5	189.0	65.0	56.1
Dielectric constant	78.54	5.02	24.60	47.00	7.52	21.01

#### Effects of extraction time, DME dosage, entrainer dosage, and entrainer ethanol:acetone ratio

Based on the data obtained in Sect. "Entrainer screening", ethanol and acetone were selected for further experiments. To study the effects of extraction time, the volume ratio of DME:microalgae:ethanol:acetone was fixed at 25:8:2:2 and extraction time was varied from 10 to 45 min. To study the effects of DME dosage, the extraction time was fixed at 45 min and the amount of DME:entrainer was varied from 25 mL per 16 mL microalgae to 25 mL per 4 mL microalgae at a constant DME:ethanol:acetone ratio of 25:2:2. To study the effects of entrainer dosage, the extraction time and ratio of DME to microalgae were fixed at 45 min and 25:8, respectively, over a range of DME:ethanol:acetone from 25:0.5:0.5 to 25:3:3. To study the effects of ethanol:acetone ratio, 8 mL of microalgae were treated with 25 mL DME:entrainer for 45 min using a total of 5 mL of entrainer (ethanol + acetone) and ethanol:acetone ratios of 1:4, 1:3, 1:2, 1:1, 2:1, 3:1, or 4:1.

### Bligh and Dyer extraction

The Bligh and Dyer method [57] is a classical method for lipid extraction, which is regarded as a reference point for determining the total lipid content of microalgae [29]. In this study, the Bligh and Dyer method was applied to both flocculation-thickened microalgae and completely dried microalgae (heated at 105 °C for 24 h). For the dried microalgae, 5.0 mL of methanol (Guaranteed Reagent, Wako Co., Ltd.), 2.5 mL of chloroform (Guaranteed Reagent, Wako Co., Ltd.), and 2.0 mL of pure water were mixed in a centrifuge tube with a dried sample that had been obtained from 10 mL of flocculation-thickened microalgae. The mixture was crushed using a homogenizer (T25, IKA Co., Ltd., Germany) at 10,000 rpm for 5 min and mixed using a Vortex Genie (SI-0236, SI Co., USA) for 5 min. Then, 2.5 mL of chloroform and 2.5 mL of pure water were added to the tube with an additional 2 min of mixing. After the sample had been centrifuged at 3,000 rpm (2410, Kubota Co., Ltd., Japan) for 5 min, it was divided into three layers from top to bottom: water–methanol, microalgae, and lipid-chloroform. Chloroform was recovered from the bottom layer; the resulting lipid-rich mixture was filtered through a 0.45-μm membrane. Most solvent was removed using a rotary evaporator at 40 °C under vacuum. The remaining solvent was removed by evaporation with a stream of nitrogen. The resulting raw lipids were weighed and stored below 0 °C. Flocculation-thickened microalgae were processed using the same modified Bligh and Dyer method, except no additional water was added. The original sample volume was 5 mL.

### Soxhlet extraction

Soxhlet extraction is commonly used to extract lipids from biomass [58]. Two solvents were evaluated in this study: hexane and hexane-ethanol (HE). For each experiment, 20 mL of flocculation-thickened microalgae were place in a cellulose extraction thimble (glass fiber, φ28 × 100 mm, Whatman) along with 100 mL hexane or 100 mL of HE (1:1 v:v). The thimble was then loaded into a Soxhlet extraction device. Each extraction was run for 24 h to ensure completeness; resulting extracts were transferred to a flask where the solvent was removed by rotary evaporation under vacuum. The extracts were then re-dissolved in 10 mL of chloroform and filtered through a 0.45-μm membrane. The solvent was then removed completely by evaporation with a stream of nitrogen. The resulting raw lipids were weighed and stored below 0 °C.

### Extract characterization

The extracted raw lipid contains not only the lipid component which is the raw material of biodiesel, but also impurities (sterols, proteins, hydrocarbons, pigments…). And the lipid component in extracted raw lipid cannot be directly quantified, it must be pretreated, and the extracted raw lipids were converted into fatty acid methyl esters (FAMEs) by transesterification. Specifically, the raw lipids were re-dissolved in 5 mL of dichloromethane (Guaranteed Reagent, Wako Co., Ltd.) and methylated by mixture with 2 mL of 14% BF_3_-methanol solution (First Grade, Wako Co., Ltd.) at 65 °C for 40 min. After the mixture had cooled to room temperature, the dichloromethane layer containing FAMEs was separated by addition of 4 mL of saturated aqueous NaCl solution (Guaranteed Reagent, Wako Co., Ltd.). After the dichloromethane layer had been isolated, the solvent was evaporated using a stream of nitrogen. The recovered FAMEs were then re-dissolved in a specified amount of dichloromethane for gas chromatography–mass spectrometry analysis (GC/MS-QP2010 Plus, Shimadzu Corp., Kyoto, Japan) using a 37-component FAME mixture (Sigma-Aldrich Japan, Co., Ltd., Tokyo, Japan) as a standard. The FAME proportions and FAME yields were calculated using the formula given below:

1 $$\mathrm{FAME proportions }\left(\mathrm{\%}\right)=\frac{\mathrm{Weight of FAMEs}}{\mathrm{Weight of raw lipid}} \times 100\%,$$

2 $$\mathrm{FAME yields }\left(\mathrm{mg}/\mathrm{g D}.\mathrm{B}.\right)=\frac{\mathrm{Weight of FAMEs}}{\mathrm{Weight of dry microalgal biomass}}$$

The carbon, hydrogen, and nitrogen contents of the extracted raw lipids were determined using an elemental analyzer (Micro Corder JM10, J-science Labo Co., Ltd.). FTIR spectra of the lipids were obtained using a Shimadzu FTIR-8400 spectrometer. Lipid samples were dropped onto a KBr tablet (Infrared spectrophotometry grade, Wako Co., Ltd.) and spectra were acquired from 400 to 4000 cm^−1^. TG and TG–DTA were performed using a Rigaku ThermoPlus TG8110 with an air flow of 100 mL/min and a heating rate of 10 °C/min from 20 to 650 °C.

Trace elements in the extracted lipids were qualitatively analyzed by inductively coupled plasma mass spectrometry (XSeries 2, Thermo Fisher Scientific Co.), and then quantitatively analyzed by inductively coupled plasma atomic emission spectroscopy (ICAP-7000, Thermo Fisher Scientific Co.). The extracted lipids (25 mg) were first digested in a mixture of 0.75 mL HNO_3_ (Guaranteed Reagent, 69%, Wako Co., Ltd.), 0.25 mL H_2_O_2_ (Guaranteed Reagent, 30%, Wako Co., Ltd.), and 15 mL H_2_O (Ultrapure Water, Wako Co., Ltd.) in a microwave digestion system (ETHOS One, Milestone Inc., USA). The mixtures were digested for 10 min at 100 °C at 500 W and then 10 min at 200 °C at 1000 W. The resulting clear digestate was collected and diluted for analysis.

### Statistical analysis

All data are presented as the mean ± standard deviation of independent experiments conducted in triplicate. Data were subjected to analysis of variance by Microsoft Office Excel 2010; differences between means were assessed by Fisher’s least significant difference test. Statistical significance was set at *p* < 0.05.

## Supplementary Information

**Additional file 1: Table S1.** The component of ESM for cultivating *Nannochloropsis oculata*. **Table S2.** The Elemental composition of extracted raw lipids by the 4 methods. **Figure S1.** Solid content of microalgae after lipid extraction by DME with 4 kinds of additive agents added. **Figure S2.** FAMEs composition of extracted lipid with 4 kinds of additive agents added, a) blank, b) ethanol, c) DMSO, d) acetone, e) THF.

## Data Availability

All data generated or analyzed during this study are included in its Additional files.
