# Supplementary material for: Effective lipid extraction from undewatered microalgae liquid using subcritical dimethyl ether
Source: Biotechnol Biofuels. 2021 Jan 9;14:17. doi: 10.1186/s13068-020-01871-0 (PMC7797121; doi:10.1186/s13068-020-01871-0)
Supplement: Supplementary file 1 — Additional file 1: Table S1. The component of ESM for cultivating Nannochloropsis oculata. Table S2. The Elemental composition of extracted raw lipids by the 4 methods. Figure S1. Solid content of microalgae after lipid extraction by DME with 4 kinds of additive agents added. Figure S2. FAMEs composition of extracted lipid with 4 kinds of additive agents added, a) blank, b) ethanol, c) DMSO, d) acetone, e) THF. [file 13068_2020_1871_MOESM1_ESM.docx]

**A novel method to extract lipid from liquid microalgae without dewatering**

Quan Wang^a^, Kazuyuki Oshita^a,^*, Masaki Takaoka^a^

^a^ Department of Environmental Engineering, Graduate School of Engineering, Kyoto University, Nishikyo-ku, Kyoto, Japan

*Corresponding author

Tel: +81-75-383-3336; Fax: +81-75-383-3338;

E-mail: oshita@epsehost.env.kyoto-u.ac.jp

Address: Cluster C, Kyoto Daigaku-Katsura, Nishikyo-ku, Kyoto 615-8540, Japan

**Table S1.** The component of ESM for cultivating *Nannochloropsis oculate*.

| **Items** | **Content** |
| --- | --- |
| NaNO_3_ | 120 mg/L |
| K_2_HPO_4_ | 5 mg/L |
| Vitamin B_12_ | 1 μg/L |
| Biotin | 1 μg/L |
| Thiamine HCl | 100 μg/L |
| Fe-EDTA | 259 μg/L |
| Mn-EDTA | 332 μg/L |
| Tris (hydroxymethyl) aminomethane | 1 g/L |
| Soil extract | 25 mL/L |
| Artificial seawater (33.4 g salts per liter) | 975 mL/L |
| pH 8.0 | |

**Table S2.** The Elemental composition of extracted raw lipids by the 4 methods.

|  | **H (%)** | **C (%)** | **N (%)** | **O (%)** |
| --- | --- | --- | --- | --- |
| **Soxhlet extraction (HE)** | 10.44±1.09 | 68.76±8.17 | 0.11±0.01 | 20.69±4.76 |
| **DME** | 11.59±0.50 | 71.09±2.70 | 0.23±0.06 | 17.09±1.59 |
| **B&D (dry)** | 9.87±2.01 | 65.52±8.10 | 0.09±0.03 | 24.52±4.82 |
| **B&D (wet)** | 12.30±0.34 | 76.21±2.22 | 0.13±0.01 | 11.36±1.30 |


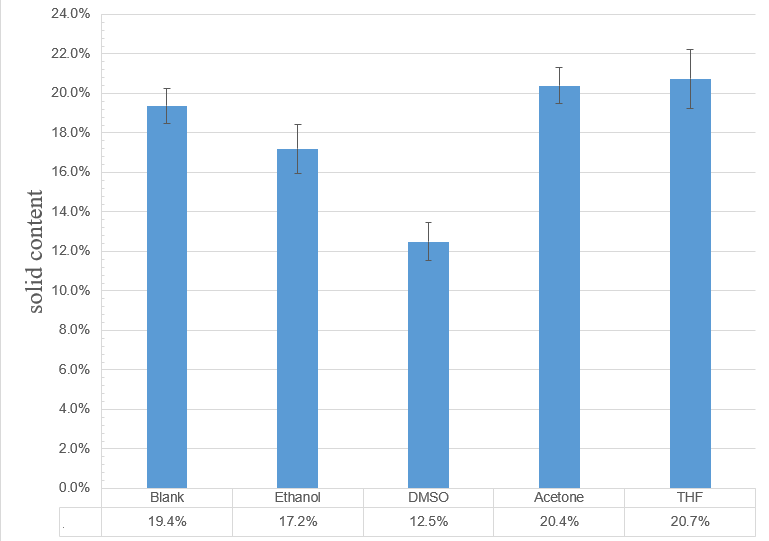


**Figure S1.** Solid content of microalgae after lipid extraction by DME with 4 kinds of additive agents added.


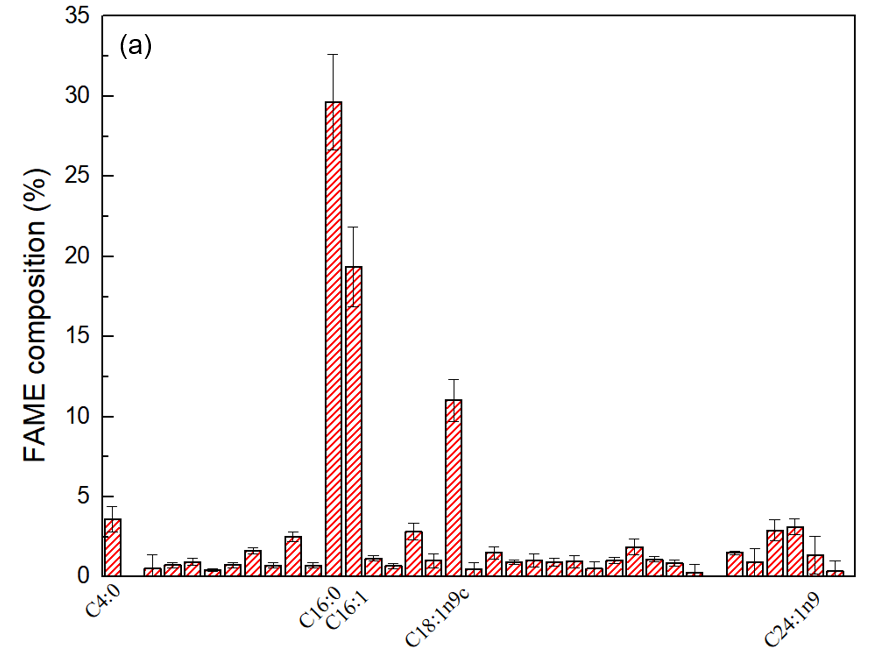


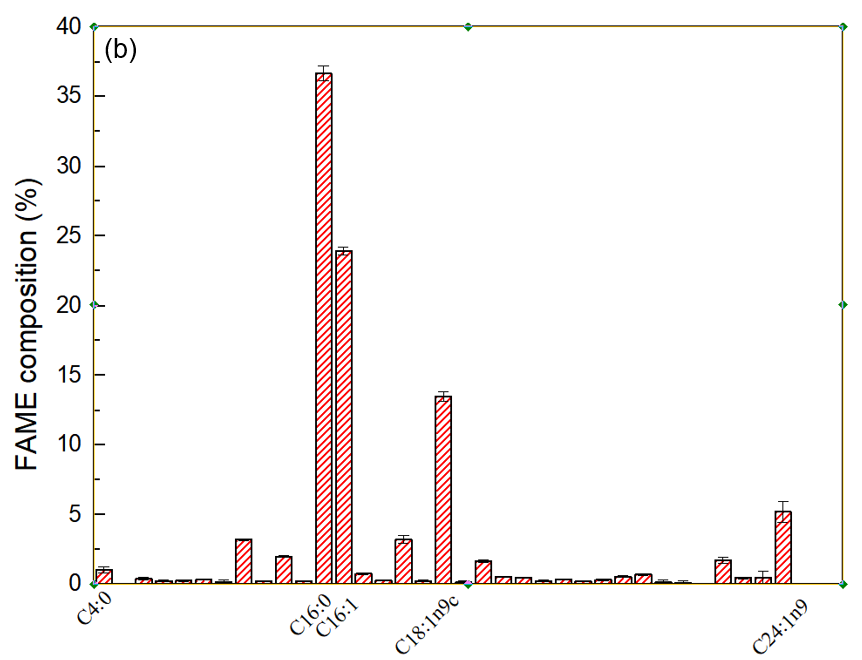


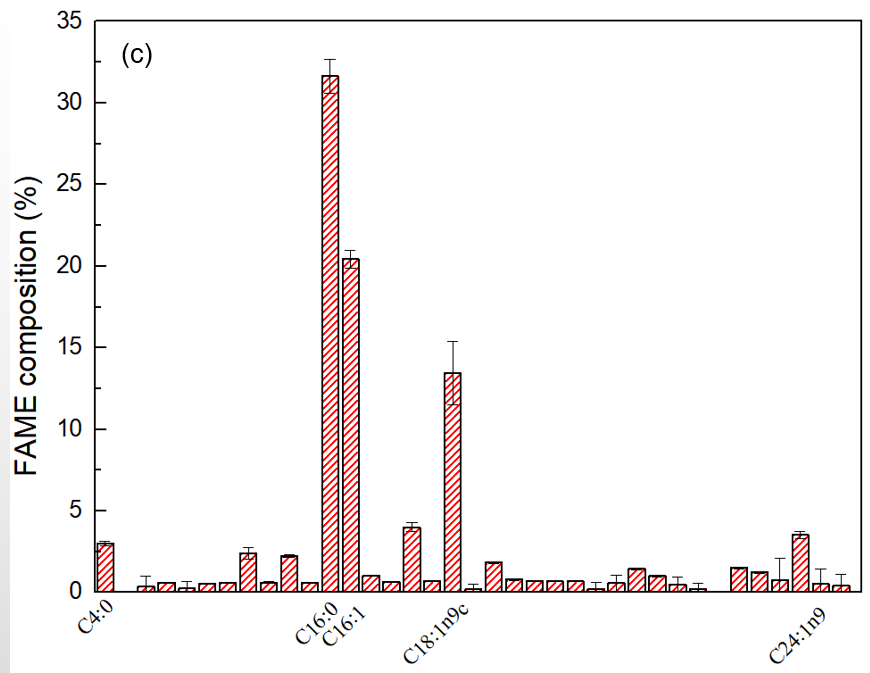


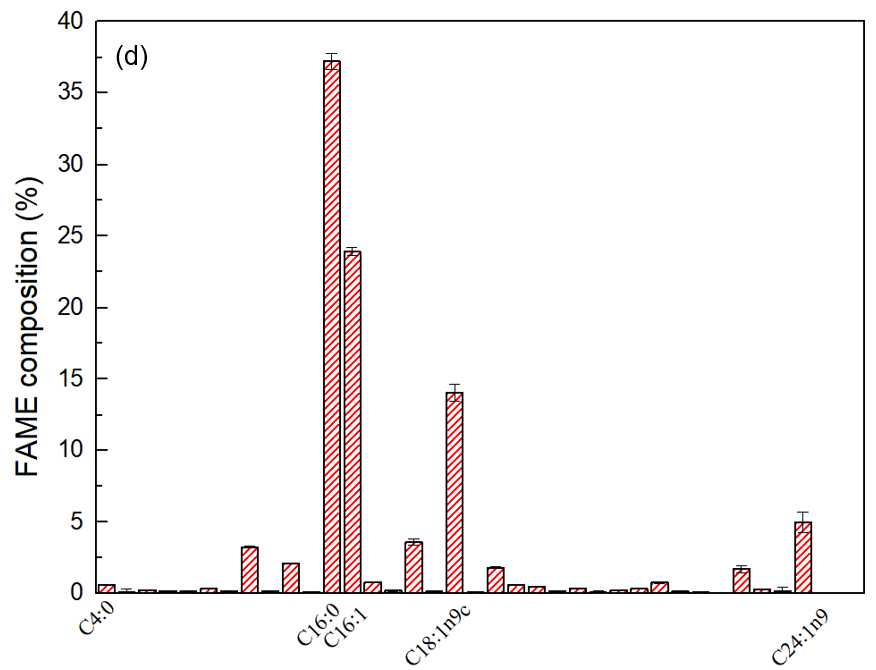


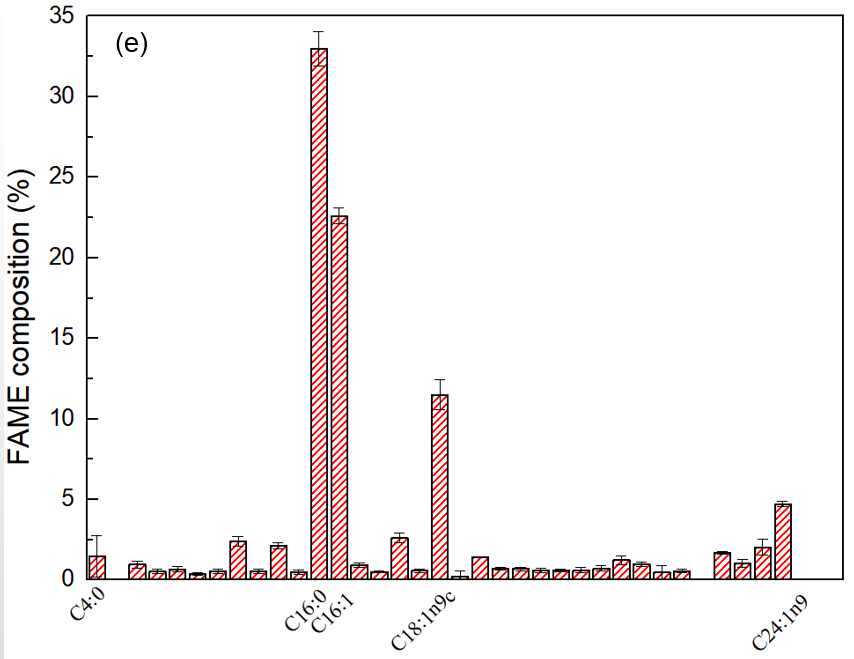


**Figure S2.** FAMEs composition of extracted lipid with 4 kinds of additive agents added, a) blank, b) ethanol, c) DMSO, d) acetone, e) THF.
